# Challenges in informed consent decision-making in Korean clinical research: A participant perspective

**DOI:** 10.1371/journal.pone.0216889

**Published:** 2019-05-23

**Authors:** Im-Soon Choi, Eun Young Choi, Iyn-Hyang Lee

**Affiliations:** 1 Department of Pharmacy, Kyungpook National University Medical Centre, Daegu, South Korea; 2 College of Pharmacy, Yeungnam University, Gyeongsan, South Korea; 3 Department of Pharmacy, Ulsan University Hospital, Ulsan, South Korea; Royal College of Surgeons in Ireland, IRELAND

## Abstract

**Objectives:**

This study investigated how the essential elements of informed consent are realised during the consent process and examined the challenges in obtaining genuine informed consent in Korea.

**Methods:**

Through purposive sampling, we recruited 21 subjects from those participating in anticancer drug research since 2013. We undertook 1:1 in-depth interviews and analysed the data by framework analysis.

**Results:**

Themes raised throughout the interviews were categorised into ‘disclosure’ and ‘understanding’ of clinical information and ‘decision’. Provider-centred information, both verbal and written, was delivered to each participant. There were few tools that the research staff might evaluate study participants’ level of understanding of the provided information during the clinical trial. Although participants did not understand basic clinical trial concepts as much as desired, they may not seek to solve difficulties through communication with trial researchers. Doubts were raised about whether participants had sufficient capacity and free will to provide informed consent.

**Conclusion:**

There is a concern that informed consent can fall short of genuine in Korea. To ensure informed consent meets the international standard, greater efforts should be made to establish an explicit standard operational protocol for obtaining informed consent.

## Introduction

During the last decade, Korea has become a major country involved in clinical research. The Korean Ministry of Food and Drug Safety reported that Korea ranked 31^st^ among global clinical trial countries in 2005, rising to 13^th^ place in 2010 and 7^th^ place in 2014 (referenced to the number of total trials) [1, 2]. This remarkable growth was fuelled by state-led policies, such as establishing criteria for clinical trials practices that were pursuant to the International Conference on Harmonisation Good Clinical Practice in 2000, introducing the Investigational New Drug application in 2002, initiating the operation of Regional Clinical Trial Centres in 2004, and launching the Korean National Enterprise for Clinical Trials in 2007. Social recognition of clinical trials has also increased; shares of Korean who were aware of the concept of clinical trial have raised 83% out of 524 persons in 2007 [3], 94% out of 137 in 2009 [4], and 97% out of 397 in 2012–2015 [5].

While medical studies of human subjects aim to benefit society, they may unavoidably raise ethical issues. Among human subject studies performed in the past, many were based on researchers’ self- regulation, resulting in studies that included bias, omission of informed consent, and invasion of privacy [6, 7]. To avoid unethical studies, international efforts have been made. Among them, the most prominent achievement is the Declaration of Helsinki that firstly announced in 1964. The latest revision of the Declaration of Helsinki was released in 2013 (7^th^) which suggested ‘respect for the individual’, ‘informed decisions’, ‘voluntarism’ and ‘subject’s welfare’ as fundamental principles, and is currently the international standard for medical research involving human subjects [8]. Current dogma regarding informed consent is set on the ethical principles of the Declaration of Helsinki. At present, researchers mandatorily obtain voluntary consent from human subjects during the initial provision of information in the first step of a clinical trial. Despite such efforts, there are concerns that, in practice, the informed consent process is executed in an expedient manner, such as “narration-followed-by-signature”, thereby losing the “informed” and “autonomous” features of informed consent. Such phenomenon may be due to participant’s resistance to medical jargon as well as their unfamiliarity with the clinical trial itself [9]. Similar concerns have been expressed that the rapid quantitative growth in clinical trials in Korea is incompatible with ethical standards [10].

In 1995, the Korean authority practically implemented Korean Good Clinical Practice (KGCP) which was firstly announced in 1987. The KCGP articles contain ethical principles that were rooted in the Declaration of Helsinki. Since then, clinical trials have been legally regulated at the ministerial order level. In 2005, ‘Hwang’s scandal [11] brought a great social impact and triggered to move related regulations to the upper law, the Pharmaceutical Affairs Act (PAA). Since 2007 clinical trials has been regulated by the PAA and its enforcement decrees. The PAA regulates trial protocol and its review process, requirements of trial institution, and researcher’s duty including ethical standard and educational training (Articles 34, 34–2 and 34–4) [12]. The KGCP requires the provision of sufficient information to the participant as a precondition of voluntary consent, and every clinical trial is required to offer each participant a well-detailed document containing 20 must- information-items (Table 1).

**Table 1:** Must contents of the informed consent document according to the Korean Good Clinical Practice, Article 7.

1	That the trial involves research.
2	The purpose of the trial.
3	The trial treatment(s) and the probability for random assignment to each treatment.
4	The trial procedures to be followed, including all invasive procedures.
5	The subject’s responsibilities.
6	Those aspects of the trial that are experimental.
7	The reasonably foreseeable risks or inconveniences to the subject and, when applicable, to an embryo, fetus, or nursing infant.
8	The reasonably expected benefits. When there is no intended clinical benefit to the subject, the subject shall be made aware of this.
9	The alternative procedure(s) or course(s) of treatment that may be available to the subject, and their important potential benefits and risks.
10	The compensation and/or treatment available to the subject in the event of trial-related injury.
11	The anticipated prorated payment, if any, to the subject for participating in the trial.
12	The anticipated expenses, if any, to the subject for participating in the trial.
13	That the subject’s participation in the trial is voluntary and that the subject may refuse to participate or withdraw from the trial, at any time, without penalty or loss of benefits to which the subject is otherwise entitled.
14	That the monitor(s), the auditor(s), the IRB/IEC, and the regulatory authority(ies) will be granted direct access to the subject’s original medical records for verification of clinical trial procedures and/or data, without violating the confidentiality of the subject, to the extent permitted by the applicable laws and regulations and that, by signing a written informed consent form, the subject or the subject’s legally acceptable representative is authorizing such access.
15	That records identifying the subject will be kept confidential and, to the extent permitted by the applicable laws and/or regulations, will not be made publicly available. If the results of the trial are published, the subject’s identity will remain confidential.
16	That the subject or the subject’s legally acceptable representative will be informed in a timely manner if information becomes available that may be relevant to the subject’s willingness to continue participation in the trial.
17	The person(s) to contact for further information regarding the trial and the rights of trial subjects, and whom to contact in the event of trial-related injury.
18	The foreseeable circumstances and/or reasons under which the subject’s participation in the trial may be terminated.
19	The expected duration of the subject’s participation in the trial.
20	The approximate number of subjects involved in the trial.

The Korean Good Clinical Practice is stipulated in the provision of Regulations on Safety of Pharmaceuticals (Ordinance of the Prime Minister) Article 30.

IRB = Institutional Review Board; IEC = Independent Ethics Committee

Informed consent documents indicate the trial’s procedural integrity and are deemed valid only after review by an Institutional Review Board (IRB) prior to the clinical trial. However, reviews by an IRB have, in reality, ended mostly as a paper review and have failed to address the question of whether research ethics were realised by conducting a 'consent procedure' with authenticity [13]. Considering the uncertainty and unpredictability of effects on the human body, which are always present in a clinical trial, the absence of a reliable, high quality informed consent procedure means that an increasingly large number of study participants in clinical trials may be exposed to unethical risks.

In Australia, Greece, the UK, and the USA, there have been multiple attempts to identify whether obtaining informed consent in clinical trials abides by related laws or regulations and whether such informed consent reflects sincere ‘voluntary consent based on sufficient information’. Example studies include factor-based evaluations of the informed consent process [14], quality evaluations of the information provided for the clinical trial [15–17], and measurement and improvement evaluations related to understanding leaflets provided to the clinical trial participant [18–22]. In Korea, few studies have explored the informed consent process. Hence, in this study, we sought to determine phenomenologically how the essential elements of informed consent are realised during the consent process and what are the challenges associated with genuine, informed consent, and decision-making in Korea.

## Materials and methods

### Study design and research ethics

We used a qualitative study design and performed 1:1 in-depth interviews consisting of open-ended questions [23]. We received the approval of the IRB of the Kyungpook National University Medical Centre (KNUMC) before undertaking data collection (Approval code: 14–1056).

### Participants

We included participants or legally authorised representatives (mostly patients’ close family), who had experienced at least once consent process in anticancer drug clinical trials from 2013 until prior to interview. Anticancer drug testing is the most frequently performed drug type tested in clinical trials in Korea, representing 157 of 607 trial approvals in 2013 (26%) and 201 of 652 trial approvals in 2014 (31%) [24, 25]. Subjects were recruited by purposive sampling, a commonly used approach in small-scale qualitative research [26]. Represents were included only if they were involved in the consent process. We excluded health professionals, illiterates, and persons with visual or hearing disabilities who might feel uneasy when interviewed.

### Themes: Disclosure, understanding, decision

The study themes were developed in consideration of previous studies. Del Carmen and Joffe defined the five components of obtaining valid informed consent as “capacity”, “decision”, “disclosure”, “understanding” and “voluntariness” [27]. In other words, to protect the study participants’ right to make a decision, the participant should be provided with full information (disclosure), should understand the given information (understanding), should be equipped with a decision-making capacity (capacity), and, based on those components, participation in the study should be determined (decision) by the participant’s free will (voluntariness). Similarly, Jones *et al*. suggested that informed consent should include three elements (disclosure, understanding and decision), and the decision would be valid when made by a capable participant without external influences [28]. A capable participant is one that is able to understand and retain the given information and to provide reasons for reaching their final decision [29]. For this study, we used *disclosure* and *understanding* of information, and *decision* as key themes. For the third theme, i.e., decision, we investigated whether participants were appropriately competent and made their decision using their free will. An interview guide was developed according to these three themes (see Supplementary information).

### Recruitment and data collection

We selected progressive, metastatic or recurrent tumour patients, or their legal represents, who had experienced at least once consent process from 2013 until prior to interview. Of those, we recruited subjects who were willing to provide their opinions. Approaching potential subjects and searching their willingness to participate was carried out in cooporation with a clinical trial nurse who was independent of this study. After explaining the research topic, objectives, data collection and recording methods, the right to decline at any time during interviews, and ensuring subjects of the confidentiality and anonymity of participation, we obtained voluntary written consent before the interview. In-depth interviews of the recruited subjects were conducted between November 1 2014 and the end of March 2015. A female doctoral student asked open-ended questions utilising the interview guide and recorded the responses. After completing each interview, the interviewer made a transcript of it and discussed each response with a female professor, PhD. Two researchers have prior experience and training in the field of qualitative study. As data collection carried on, questions were slightly modified to encourage participants to express their opinion more freely and frankly, or to add sub-themes that needed further consideration. Interview time ranged from 40 minutes to 1 hour 15 minutes, with median of 45 minutes (average 48.5 minutes). Interviews took place at a comfortable, independent office in the KNUMC. Recruiting of study participants was terminated when we observed theoretical saturation, i.e., when few new issues were raised and/or when the same types of concepts and topics were being repeated [30].

### Data analysis

Collected data were analysed by using framework analysis, which comprises five steps [31]. The interviewer conducted the first step of framework analysis and then extracted the themes in subsequent steps 2–5 via continuous cross-checking and discussion with two other researchers. The whole process progressed as follows. First, as a familiarisation step, the interviewer repeatedly listened to the recorded interview files and transcribed them into text. Participant identity was hidden by numbering the data file and removing personal information during transcription. Second, categories were derived by identifying emerging ideas, feelings and concepts, and a short note was added next to the text, forming a descriptive statement. Inductive thematic coding with repeated words or specific meaning during each interview was applied. Third and fourth, an indexing and charting process was undertaken in which data were moved and quotes were organised, highlighted and compared within one case or between cases. Fifth, topics, subtopics and details were extracted after mapping and interpreting. Phrases quoted in the results were translated to English by an independent native English speaker. The English phrases were then translated back to Korean to be cross-checked to determine whether the meaning was delivered properly. When a representative response was quoted, a personalised serial number was assigned, and the line number of the transcript of the statements was written (e.g., I12:25–26 corresponds to line numbers 25 and 26 in a conversation with I12 interviewee).

## Results

### Participants’ characteristics

There were 21 study subjects, including 10 clinical trial patients and 11 represents. Age distribution of the participants was 30–70 years with participants in their 50s being most common (43%). Participants with high school education or higher represented 81% of the total. Fifteen (71%) of the participants were associated with breast cancer trials. The disease of each cancer subject was classified according to the Tumour-Node-Metastasis staging system enacted by the American joint committee on cancer. Six participants (28.6%) were associated with stage 3 cancer and seven (33.3%) were associated with stage 4 or metastatic cancer. Nine participants (42.9%) had prior experience with a different anticancer therapy before their current clinical trial, whereas 20 (95.2%) were enrolled in a clinical trial for the first time. Seventeen (81%) were in a phase 3 clinical trial and 13 (61.9%) were in a randomised double-blind trial. Other demographic characteristics are summarised in Table 2.

**Table 2:** Participants’ demographics.

Characteristic		N (%)
Participant	Patient Representative	10 (47.6%) 11 (52.4%)
Sex	Men Women	10 (47.6%) 11 (52.4%)
Age (years)	30–39	4 (19.0%)
	40–49	4 (19.0%)
	50–59	9 (42.9%)
	60–69	3 (14.3%)
	70 or older	1 (4.8%)
Education degree	Elementary school or below	2 (9.5%)
	Middle school	2 (9.5%)
	High school	8 (38.1%)
	University or above	9 (42.9%)
Diagnosis	Breast cancer	15 (71.4%)
	Gastric cancer	3 (14.3%)
	Pancreatic cancer	2 (9.5%)
	Melanoma	1 (4.8%)
Disease status (TNM stage)	Stage I	2 (9.5%)
	Stage II	6 (28.6%)
	Stage III	6 (28.6%)
	Stage IV	7 (33.3%)
Chemotherapy prior to clinical study	Yes	9 (42.9%)
	No	12 (57.1%)
Previous experience in clinical trials	Yes	1 (4.8%)
	No	20 (95.2%)
Trial phase	Phase II	4 (19.0%)
	Phase III	17 (81.0%)
Study design	Randomised, double-blind	13 (61.9%)
	Randomised, open-label	6 (28.6%)
	Non-random, open-label, single arm	2 (9.5%)

### Information disclosure

#### The process

Information transmission between the trial researchers (including doctors and other staff) and the trial participants mainly occurred in face-to-face verbal exchanges. Along with verbal communication, written information was provided in the form of a combined document comprising a participant information leaflet and a consent form. Participants were provided such documents in different situations. Although the documents were provided mostly at the same time as the verbal study description, they were sometimes provided after receiving verbal consent. *“Did I have to read information sheets before I decided to participate*? *But those were provided after I made a decision*, *so I was dissatisfied (I13*:*150–151)*.*”* Participants’ statements raised doubts about whether the documents conveyed information for consent at an appropriate time. For example, knowledge of possible adverse effects could become known after giving consent rather than during the consent discussion. *“It is desirable for doctors to give the participant instructions regarding side effects of pills not during the treatment procedure but from the initiation (I3*:*199–200)*.*”*

#### The contents

The study participants frequently commented ‘I do not remember,’ when asked to explain some clinical terms, processes, *etc*. Despite the provision of written information, the majority of participants had difficulties to understand that information, saying *“It was hard to read the densely written form of over ten A4-sized pages and I could catch only 10% of the entire contents (I16*:*168–169)”* or *“Terms have never been heard (I7*: *90–91)*.*”*

There were conflicting opinions about the provided information documents. Some demanded that the information should be expanded so that it explained professional medical terms, while others insisted that the large amount of information should be shortened. One participant suggested some methods to improve the information provided to laypersons. *“The leaflet looks reserved only for those who are professional*. *It would be helpful to understand with photos and clear mark of the test’s objective (I16*:*184–185)*.*”*

### Understanding

As Table 3 shows, of the main information items that should be delivered to participants in clinical trials, the majority could not be understood or the content was misunderstood. For example, participants seldom understood voluntary withdrawal, rarely knew what kinds of alternate options they were able to choose, or they falsely understood the definition of randomisation. They also suspected that *“since the clinical trials proceed step by step to ensure the effectiveness of the drug*, *therapeutic effect will appear slowly (I18*:*56–57)*,*”* or *“since clinical drugs are free of charge*, *the effects may be worse (I20*:*74–77)*.*”* Furthermore, the disclaimer clause of the tested drug sponsor, regarding the exemption of compensation for unrelated damage, overwhelmed participants with concerns about their unprotected situation after they decided to participate. *“I was too troubled by the clause of ‘~ have no responsibility’ in the leaflet to be anxious about my decision to participate (I16*:*172–174)*.*”*

**Table 3:** Understanding.

Sub-theme	Participants’ statements
Voluntary participation and withdrawal	*“I can quit whenever I want to stop to participate in the clinical trial*.*” (I2*:*104–105)* *“I have never thought of voluntary stop*. *For now*, *I am only thinking about how long it may take*, *I have to do*.*” (I19*:*116–117)*
Random allocation	*“The doctor said nobody knows which pill I would take*: *either a placebo or a real drug*.*” (I12*:*39*, *101)* *“It is wrong to randomly allocate and only doctors have the right of decision*. *With no medical knowledge*, *the patient cannot decide what drugs to take*.*” (I18*:*76–78)*
Alternative procedure	*“I was informed to add clinical drugs onto the conventional treatment*. *Excluding the clinical pills*, *the procedure would go on with the conventional treatment*.*” (I1*:*24–26)* *“Since I wanted to participate in the clinical trial from the first*, *I had never thought of being informed of other treatments*.*” (I9*:*42–43)* *“I cannot remember even whether I heard of it or not*. *I read the leaflet thoroughly*, *however*, *cannot remember what the next step was in case of no participation in the clinical trial*.*” (I10*:*134–136)*
Clinical trial phase	*“Phase 3 trials include full fix of side effects*.*” (I2*:*85)* *“Since the clinical trial is done after animal experiments*, *there are no serious injuries for humans*.*” (I6*:*85–86)* *“Phase 1 trial is insufficient to come into the market*. *To my knowledge*, *the clinical drugs are for sale after phase 3 trials and verification*.*” (I19*:*148–149)*
Potential risks	*“It is blessing for the improvement as a good result of clinical trials*, *however*, *for no significant effect*, *I would regret for having participated in the test and missed the right time of treatment*.*” (I5*:*104–105*, *127)* *“Since I have no knowledge of the trial drug*, *I am concerned about what side effects would occur*.*” (I16*:*66)* *“I was concerned if I would go wrong during the test and felt being somewhat like a subject of test*.*” (I21*:*136–137)*
Period and method of treatment	*“I was informed about the duration of participation*. *However*, *it is not meaningful for the patient since I should take drugs as long as I can*.*” (I6*:*47–48)*
Degree of understanding about written information	*“It is not difficult to understand the content of the leaflet*. *Since I already made my mind to participate in the test*, *I easily accept the way they wrote*.*” (I10*:*170*,*173)* *“In fact*, *I could not fully understand with explanation given by a nurse*. *However*, *the leaflet was conducive for me to understand what test it was*.*” (I21*:*91–92)*
Willingness to use written information	*“I often refer to the part of ‘Adverse Reactions’*.*” (I2*:*167)* *“Since I already fully agreed to participate in the trial*, *I rarely give a significant meaning to the leaflet and the consent*.*” (I12*:*137–138)* *“I kept the leaflet since I had thought the evidence data would be needed in case of an abnormality*.*” (I17*:*193)* *“Considering it as a mere process*, *I tended to browse only headlines and pass medical terms*.*” (I18*:*123)*

When being asked relevant issues, participants often answered that 'it was not explained' and indicated that they were not fully aware of the procedure, such as *“though I hope to stop when the patient is suffering*, *I don't know how to do (I11*:*56–57)*.*”*

One participant strongly addressed *“it was better to proceed with checking how much the patients understand rather than to let them simply read*. *I was stuffy when it simply went through (I6*:*268–271)*.*”*

Throughout interviews, therapeutic misconception was observed. Although participants were aware that clinical trials were purposed for medical research, the majority of them actually put weight on the treatment of the associated disease. *“I was involved for treatment because I felt like clutching at a straw (I11*:*25)*.*” “Given that my disease is incurable with current drugs*, *the doctor recommended this to me*, *saying it is a good drug (I19*:*81–83)*.*”* There were participants who tended to remember only the hopeful part, such as saying *"clinical drugs are the best treatment (I20*: *99)*,*"* or recognised that phase 3 clinical trials were safe because adverse reactions were mostly removed (Table 3).

### Decision

Participants who fully understood the given information and used that understanding to deal with a lack of knowledge over clinical research or to appreciate the benefits and risks they would experience after participation were uncommon. The study participants seemingly made decisions to participate under the perception that the trial was the last resort of treatment, rather than deciding as a result of careful consent discussion. Many were likely to make decisions that depended on the opinions of family members or friends, or on referring to the internet for information, rather than on personally understanding the contents of the clinical trials and gaining proper knowledge. *“I hesitated a lot*. *I made a decision*, *not by the leaflet*, *but asked to relatives whether I should participate in or not (I20*:*154–155)*.*”* Also, as stated, understanding by subjects was far from a level adequate for them to weigh the benefits and risks of the clinical research. Sometimes they blindly trusted the medical staff. *“I don't need the leaflets because I think doctors will take care of all that*, *I think doctors do the clinical trials to treat patients in a better way (I2*:*126–130)*.*” “The professor (doctor) who recommended me to participate would guarantee the use of the best drug and speedier care decisions (I15*:*164–165)*.*”* In addition, participants often expressed satisfaction with their situation of being treated differently, *“unlike other cases*, *doctors kindly informed me regarding the clinical trial (I15*:*46–47)*.*”*

To make matters worse, study participants often displayed little will to gain knowledge from the given information. For example, participants had high concerns and fears about side effects, but they were reluctant to ask questions about matters of which they were uncertain. Time pressure was one of the key reasons for that reluctance, *“we can see docs in just 2~3 minutes… There are also other patients waiting behind (I18*:*64–65)*.*”* More often, however, participants stated the reasons for their passivity were *“our position is to believe and just follow (I6*:*15)*,*” “will the results be different by asking a question*? *(I17*:*225)”* so they may feel *“the manual or consent are generally thought to be signed and that’s enough… (I9*:*191)*.*”*

The biggest concern was raised by a statement questioning whether study participants actually possessed the freedom to reverse the decision to participate. *“I made a decision to participate in clinical trials*, *but I thought I didn't want to do as I feel a little worrisome after thinking about it at home*. *But at the next visit*, *I was told I had to participate because it has already been decided (I5*:*62–66)*.*”*

## Discussion

The clinical trial industry in Korea has grown rapidly, which means that an increasing number of people may be exposed to risks related to clinical trials. Thus, obtaining genuine informed consent process is more and more important in order to validate clinical research ethically and legally. This study aimed to explore challenges associated with informed consent decision-making in Korea from a clinical trial participant perspective. The results show that Korean patients seem to make decisions to participate in clinical research without external pressure, coercion, or manipulation. However, we observed various phenomena that brought us to question whether a trial participant’s handwritten signature can be accepted as a true indication of a voluntarily made, genuine informed consent.

We identified the following matters as challenges in informed consent decision-making in Korean clinical research. There was concern that patient involvement in the decision-making process may be affected by the composite actions of a patriarchal patient-medical paradigm and family-based culture. Patients’ autonomy were frequently concealed by family determinants. Moreover, they showed strong dependence tendencies on medical staff in the consent process, which was far from that present in ‘a therapeutic alliance’ [28] or ‘a mutual relationship’ [32]. It may be associated to the Confucian culture that values collective decisions higher than individual ones [33], or to a passive understanding of autonomy in the Korean society [34].

Throughout interviews, it was uncertain whether the order of ‘information provision–consideration–participation decision’ was strictly followed. In fact, the real situation is that the time to provide written information varies and can occur anytime between the introductory dialogue and the time following the signing of a consent form; moreover, the cooling-off period after consent was undefined. Adequate attention to providing a proper time for deliberation was not commonly reported by our participants.

Verbal information was provided by trial researchers through a 1:1 dialogue as a core requirement for obtaining informed consent in Korean clinical trials. The use of this approach is supported by a systematic review which suggested that the most effective way to realise informed consent might be to provide a 1:1 sufficient dialogue between researchers and participants [18]. However, whether it actually worked as expected was in question, as the time duration for mutual communication of study information and the depth of that discussion prior to requesting informed consent may be insufficient for participants to understand the information and make a decision. In general, participants’ understanding of the essential points of the clinical trial was below that expected. Medical information is a field in which informational asymmetry between providers and recipients is significant. According to a recent Korean study, approximately 45% of 104 hospitalised patients did not properly understand the explanation provided by a doctor [35]. Parsons demonstrated that the ordinary patients in clinical studies, no matter how high the level of their education was, could not accurately understand the medical information, and often experienced psychological withering which reduced their capacity to understand information [36].

The importance of written information in the consent process should be stressed. They are forms of persistent data and legal documents that can overcome the weakness of a verbal description. Written information is particularly important for participants with difficulties like short-term memory loss [37]. In this study, we found that written information was distributed to participants, but it consisted of structures and vocabularies that might not consider the health literacy of participants. According to a study performed recently in Korea, informed consent documents include 15 pages and 6,245 Korean words on average [38]. It is concerning that such a long document may not be delivered effectively as this document size is far greater than that suggested by Sharp (a maximum of five pages with 1,250 English words which is suitable for regular adults to read in 5–7 minutes) [39]. In addition, clinical trials sponsored by multinational pharmaceutical companies are likely to use expressions that are difficult to translate into Korean, which could decrease further the participants’ motivation to use the provided information leaflets.

Although it was observed that an inaccurate understanding of clinical trials can produce problematic situations, the will of the participants to increase their understanding was notably low. Participants hesitated to ask questions and sometimes seemed to think that asking questions would be useless. Moreover, they might have psychological barriers in participating in a horizontal communication with a doctor. In addition, participants seemed to have little intention to utilise the provided written information. They seldom used the provided leaflets to seek information for making their decision but did make use of them for determining visiting schedules. A recent study argued that the tendency of study participants not to read the leaflet was partly due to the asymmetrical relationship between researchers and participants [40]. On that basis, it may be crucial that information providers evaluate information receivers to determine how much they understand the presented information. At present, there are no provisions for letting trial researchers consider the level of understanding of participants in the consent process.

According to a meta-analysis of understanding of the informed consent of participants over the last 30 years, the proportion of patients who understand the items associated with informed consent ranged from 52% to 76%, and at least a quarter and up to about half of clinical trial participants might have an incorrect understanding of the trial [41]. In particular, participants had low levels of understanding of the potential risks associated with adverse reactions, placebo use and freedom to withdraw from the trial. The items being associated with low understanding in Tam *et al*. [41] were similar to those in our results, indicating that little has changed for 30 years. Thus, if patients base their decisions on superficial information and a low understanding of clinical trials, they may have the misguided expectation that clinical trials can provide a better treatment than that from a conventional approach. A survey of cancer patients showed that 46% of respondents recognised clinical drugs as the best treatment choice or as a cutting-edge technology [3]. While the participants in the present study reasonably agreed that a clinical trial was research with experimental significance associated with uncertainty rather than with treatment, most of them had a therapeutic misconception about their own trial, as has been previously reported [42–44]. Many participants were optimistic about the safety of clinical drugs or phase 3 clinical trials; however, there was a considerable gap between their optimistic perception and the real success rates of clinical trials with phase 2 success rates of 18%-28% and phase 3 success rates at 50% or less [45, 46].

### What should be improved in order to establish genuine informed consent?

First, we recognise the necessity of establishing a standard operation guide that would quantitatively indicate the duration of the one-on-one oral interview, the timing of providing written information and the consideration time for a final decision. Korean good clinical practice now regulates the provision of ‘sufficient opportunities’ and ‘time’; however, this study shows that more detailed guidance is needed to protect participants. There are some examples related to protecting patients’ autonomy; for example, in an out-patient clinic in Copenhagen, the minimum time of consideration for the patient to participate after obtaining full understanding is 24 hours [47]. In a university hospital in Norway, the procedure requires confirming a patient’s decision to participate at 24 hours after the initial consent [48].

Second, it is necessary to monitor regularly whether the information documents for a clinical trial are provided in a patient-centred manner. For genuine consent, evaluation of the participants’ continued comprehension of the given information during the entire period of participation is needed [49]. In addition, there is a combined need to evaluate whether the structure and the content of the leaflet meets the understanding level of participants. In addition, it may be worthwhile verifying the availability and readability of the information leaflet directly among consumers by using performance-based user testing [50–52].

Third, with a long-term perspective, a social effort for improving participants’ decision-making abilities is needed. As long as the participants’ understanding or willingness to understand stays at the low level reported in this study, it will be difficult to obtain truly informed consent only by relying on trial researcher sincerity and institutional propriety.

### Strengths and weaknesses

This study has two important strengths. First, it is the first qualitative study in Korea based on data collected from actual participants in clinical trials who went through the consent process. Previous studies on this topic in Korea used quantitative methods [53, 54]. Second, this study is different from other previous studies as it specifically explored the basic aspects of the clinical trial consent process and analysed participants’ understanding of the information provided at the time of participation.

Assessment of this study also requires consideration of its limitations. First, we recruited participants who took part in anticancer drug clinical trials at a single hospital. Since the detailed method and process in the course of obtaining consent could differ by institution and by disease characteristics (e.g., cancer patients might be in different situations than participants with other diseases), these may limit the ability to generalise from the results of this study. Second, we entirely depended upon the participants’ statements obtained via recall and did not observe the consent process in person, which was beyond the scope of this study. Therefore, it was impossible to observe the trial researchers’ communication skills and other aspects of the consent process. Our results may be affected by several factors such as participants’ selective acceptance of information or paramnesia. To overcome this limitation in a future study, we suggest that direct observation of the consent process be undertaken. Third, since most of the participants took part in clinical trials for the first time, it was probable that they lacked opinions and experiences that would have arisen from prior enrolment. A large number of the participants reported a positive experience when participating in their clinical trials, leading to stating favourable opinions and the potential risk of selection bias.

## Supporting information

S1 File Interview guide: Korean. (DOCX)

S2 File Interview guide: English. (DOCX)
