# Supplementary material for: Challenges in informed consent decision-making in Korean clinical research: A participant perspective
Source: PLoS One. 2019 May 23;14(5):e0216889. doi: 10.1371/journal.pone.0216889 (PMC6532870; doi:10.1371/journal.pone.0216889)
Supplement: S1 File — (DOCX) [file pone.0216889.s001.docx]

**인터뷰 가이드**

**I. 정보제공**

**1. 설명의무**

*a. 임상시험 참여동의는 어떤 절차를 통해 이루어졌는지 설명해 주시겠어요?*

*b. 동의절차 중 의문점이 생기면 어떻게 해결하셨습니까?*

**2. 정보의 충분성**

*a. 임상시험에 대한 설명을 받은 후 결정할 때까지 시간이 얼마나 소요되었나요?*

*b. 참여 시 모르거나 궁금한 사항은 모두 해결하셨나요?*

*c. 임상시험 참여가 어떤 장점이 있다고 생각하셨나요? 임상시험 참여가 어떤 위험을 가져올 수 있다고 생각하셨나요?*

**3. 시험대상자설명서**

*a. 이것은 샘플인데요(시험대상자설명서), 받으신 설명서와 비슷한가요? 받으신 설명서에 대해 좀 더 설명해 주시겠어요?*

*b. 시험대상설명서를 제공하는 이유가 무엇이라 생각하세요?*

*c. 참여 시 가장 궁금한 것은 무엇이었나요?*

**II. 연구참여자의 이해**

**1. 임상시험에 대한 기본 지식**

*a. 임상시험이 무엇인지 생각나는 대로 말씀해 주시겠습니까?*

*b. 평소 임상시험을 하는 목적이 무엇이라고 생각하셨습니까? 왜 임상시험이 필요하다고 생각하십니까?*

**2. 참여하는 임상시험에 대한 이해**

*a. 참여한 임상시험의 목적은 무엇이었습니까?*

*b. 참여한 임상시험의 치료방법과 치료기간에 대해 설명해 주시겠습니까?*

*c. 참여하지 않을 경우 선택할 수 있었던 다른 치료법에 대해 설명해 주시겠습니까?*

*d. 임의배정에 대한 내용을 설명해 주실 수 있으신가요?*

*e. 참여한 임상시험약이 어느 단계의 개발 약인지 설명해 주실 수 있으신가요?*

*f. 임상시험을 중단하고 싶을 때 어떻게 하실 것인지 말씀해 주시겠습니까?*

*g. 임상시험에 참여함으로써 얻게 되는 이익이나 위험 또는 불편감은 무엇인지 설명해 주시겠습니까?*

**3. 시험대상자설명서에 대한 이해**

*a. 설명서와 동의서 보관은 어떻게 하고 계십니까?*

*b. 시험대상자설명서를 어떻게 활용하고 계십니까? 시험대상자설명서의 어느 부분을 가장 자주 보셨는지요? 그 이유는 무엇인가요?*

*c. 시험대상자설명서 중 이해가 어려운 부분은 어떤 것이었습니까?*

*d. 이해하지 못한 부분은 어떻게 해결 하셨는지요?*

*e. 받으신 시험대상자설명서에서 내용에 대한 이해도를 높이기 위해 개선이 필요한 부분이 있다면 말씀해 주시겠어요?*

**III. 자기결정권 발휘**

**1. 자발적 의사결정 의지**

*a. 임상시험에는 왜 참여하기로 결정하셨나요?*

*b. 임상시험 참여는 혼자 결정 하셨나요? 혹시 함께 의논한 분이 있으셨나요?*

*c. 참여결정 전 임상시험설명서 외에 찾아본 자료가 있었다면 설명해 주시겠어요?*

**2. 자기결정권에 대한 간섭요인**

*a. 임상시험 참여를 결정하는데 도움이 된 사람/의견/정보/환경/경험 등이 있으시면 설명해 주시겠어요?*

*b. 임상시험 참여를 결정하는 것에 장애가 된 사람/의견/정보/환경/경험 등이 있으시면 설명해 주시겠어요?*

**3. 의사결정에서의 시험대상자설명서**

*a. 임상시험 참여결정 시에 시험대상자설명서는 어떻게 활용하셨는지요?*

*b. 시험대상자설명서 중 임상시험 참여의사를 결정하실 때 가장 많이 보신 부분이 무엇인지요?*

*c. 임상시험 참여를 결정하는 데 도움이 되기 위해 시험대상자설명서가 개선되어야 할 부분이 있다면 말씀해 주세요.*
