# Supplementary material for: Challenges in informed consent decision-making in Korean clinical research: A participant perspective
Source: PLoS One. 2019 May 23;14(5):e0216889. doi: 10.1371/journal.pone.0216889 (PMC6532870; doi:10.1371/journal.pone.0216889)
Supplement: S2 File — (DOCX) [file pone.0216889.s002.docx]

**Interview Guide**

**I. Information provision**

**1. Duty to explain**

*a. Could you describe the process through which you gave your consent to participate in the clinical trial?*

*b. How did you resolve any questions that you had during the consent process?*

**2. Sufficiency of information**

*a. How long did it take for you to make a decision to participate after being explained the trial details?*

*b. At the time of participation, were you able to resolve all of the questions that you had?*

*c. What benefits/potential risks did you think you could gain/bear by participating in the clinical trial?*

**3. Participant information sheet (PIS)**

*a. (Showing an example PIS.) Is it similar to the PIS you received? Please explain yours more.*

*b. Why do you think patient information sheets are provided?*

*c. What was your biggest concern when you participated?*

**II. Participant's understanding**

**1. Basic knowledge of clinical trials**

*a. Could you explain what a clinical trial is (as much as you can)?*

*b. What do you think the purposes of clinical trials are? Why do you think we need clinical trials?*

**2. Understanding about the clinical trial in which the individual is participating**

*a. What was the purpose of the clinical trial you participated in?*

*b. Could you describe the treatment methods and duration you received in the clinical trial?*

*c. Could you explain what alternative treatment available for you, if you had decided not to participate in the trial?*

*d. Could you explain the concept of randomization?*

*e. Would you know what phase your trial drug was in?*

*f. Could you explain what you would do if you don’t want to take part in your clinical trial anymore?*

*g. Could you explain any benefits, risks or discomfort you may get in your clinical trial?*

**3. Understanding about the participant information sheet which the individual is provided**

*a. How have you kept the PIS and consent form?*

*b. How have you utilized the PIS? Which section of the PIS have you read the most and why?*

*c. Which section of the PIS would you find difficult to understand?*

*d. How have you coped with anything you cannot understand?*

*e. Would you have any suggestions for a PIS to improve general participants’ comprehension?*

**III. Self-determination**

**1. Ability to make voluntary decisions**

*a. What makes you to decide to take part in the clinical trial?*

*b. Did you make a decision to take part in the trial on your own? Did you discuss it with anyone else?*

*c. Before making the decision, did you look up any other sources of information other than the PIS? If so, please describe what they were?*

**2. Factors influencing decision making**

*a. Could you describe which persons/opinions/information/environmental factors/ experiences helped you decide to participate in the trial, if any?*

*b. Could you describe which persons/opinions/information/environmental factors/ experiences made you hesitate to join in the trial, if any?*

**3. Participant information sheet in decision making**

*a. How did you utilize the PIS when you made your decision to participate in the trial?*

*b. Which section of the PIS did you read the most when you made your decision to participate in the trial?*

*c. Would you have any suggestions for a PIS to help general participants’ decision making?*
